# Development of a Prognostic Score in Patients With Advanced Breast Cancer Treated for Meningeal Carcinomatosis

**DOI:** 10.1155/tbj/5438600

**Published:** 2025-03-07

**Authors:** Grégoire Narjoux, Juliette Mainguené, Marie-Noëlle Guilhaume, Olivier Brenet, Edith Borcoman, Laurence Escalup, Hélène Salaun, Pauline Moreau, Anne-Sophie Bouyer, Paul Cottu

**Affiliations:** ^1^Department of Medical Oncology, Institut Curie, 26 rue d'Ulm, Paris 75005, France; ^2^Department of Anesthesiology, Institut Curie, 26 rue d'Ulm, Paris 75005, France; ^3^Pharmacy, Institut Curie, 26 rue d'Ulm, Paris 75005, France; ^4^Université Paris Cité, Paris, France

**Keywords:** breast cancer, Cyfra 21-1, meningeal carcinomatosis, prognostic score

## Abstract

**Purpose:** Meningeal carcinomatosis (MC) has a dismal prognosis in patients with breast cancer and requires invasive therapies. The aim of the present retrospective study was to determine a prognostic score for overall survival (OS) in patients with breast cancer and treated for MC.

**Methods:** The data of 109 patients with proven breast cancer MC treated with at least one intrathecal (IT) injection of methotrexate or thiotepa at Institut Curie were retrospectively recorded from 2011 to 2019. We developed prognostic clinical scores for OS and 24-week survival.

**Results:** The diagnosis and evaluation of MC were based on a combination of clinical, imaging, and laboratory studies. Three significant prognostic factors for OS were identified. Clinical response or stabilization after one month of IT therapy had a favorable independent prognostic value for both OS and 24-week survival. Additionally, a baseline CSF Cyfra 21-1 concentration lower than 79 ng/mL in the cerebrospinal fluid (CSF) and the absence of 1-month CSF malignant cells had borderline favorable independent prognostic value for OS and 24-week survival, respectively. We constructed 2-class and 3-class prognostic scores for each outcome, identifying a population with a very poor prognosis.

**Conclusions:** To our knowledge, this is the first study to develop a response-based prognosis score for patients with breast cancer-related MC. This one-month prognostic score may help to determine which patient could actually benefit from prolonged IT therapy.

## 1. Introduction

Meningeal carcinomatosis (MC) is defined as leptomeningeal infiltration of tumor cells, including the pia mater, arachnoid, and subarachnoid spaces, from a primary malignant tumor. Breast cancer (BC) is the most common etiology of MC among solid tumors, and MC occurs in approximately 5% of patients with metastatic BC [1, 2], who subsequently have an extremely poor prognosis [3, 4].

The gold standard for the diagnosis of MC is cerebrospinal fluid (CSF) cytology, but this technique has poor sensitivity [5]. Imaging (mostly MRI) and elevated protein levels in the CSF strongly suggest a diagnosis without acknowledged standards [6], and there is neither a validated assessment tool nor a validated assessment tool during the course of MC [7]. Our group suggested that elevated Cyfra 21-1 in the CSF is associated with an MC diagnosis and has potential prognostic value [3]. There is also no consensus about treatment, and treatment effectiveness is low [8]. At our institution, patients with BC MCs are generally treated with a dose-dense intrathecal (IT) methotrexate regimen in combination with other systemic treatments whenever possible [3]. There are no generally accepted criteria defining patient subgroups who might benefit from IT therapy [8], and patients have often been heavily pretreated with various systemic drugs with few remaining therapeutic options.

Here, we report our updated experience in patients with advanced BC and MC. We previously developed a poorly validated 4-parameter prognostic score based solely on baseline clinical features, which did not take into account the early clinical evolution in treated patients [8, 9]. The aim of the present retrospective study was to determine a prognostic score for overall survival (OS) *in patients with BC-related MC who were initially considered amenable to IT therapy* to provide a relevant tool to help clinicians decide who might benefit from continuation of invasive treatment regimens.

## 2. Patients and Methods

We retrospectively recorded all patients with proven BC-related MC who were treated at least once with IT from 2011 to 2019. The key eligibility criteria were histologically proven breast carcinoma; MC as defined either by tumor cells in the CSF or a combination of neurological symptoms and/or compatible radiological results (CT scanner or MRI) and/or elevated total protein or Cyfra 21-1 levels in the CSF; and treatment with at least one IT injection of methotrexate or thiotepa. Patients who received IT therapy were identified through a systematic search of the electronic records of the hospital's chemotherapy database. All the electronic medical records of the identified patients were manually analyzed, and details on MC diagnosis, treatment, and clinical course, including CSF Cyfra 21-1 level, CSF protein count, and CSF malignant cells that were collected at diagnosis and after one month of IT treatment, were collected.

### 2.1. Statistics

The principal objective of the study was to determine a prognostic score for OS in patients with newly diagnosed MC who were considered amenable to IT therapy. Conventional descriptive statistics were used. OS was defined as the time elapsed from MC diagnosis until death from any cause. OS prediction curves were constructed with the Kaplan–Meier proportional hazard model. Multivariate analyses were performed with Cox proportional hazards regression, with death as the endpoint and using a forward variable selection procedure, based on the results of the univariate analyses. Final estimates with a *p* value (Wald) < 0.05 were retained. Due to the very poor prognosis of patients with MC, we refined the analyses by focusing on 24-week OS. Based on previous reports [8–10], we considered the following variables of interest, all related to BC history and symptoms of MC, in the univariate and multivariate analyses: pathological subtype (triple negative (TN) vs. other), clinical presentation (visceral metastases and neurological symptoms), previous therapies, CSF protein level (abnormal vs. normal), and CSF Cyfra 21-1 levels with various exploratory threshold values. Based on our previous report [9], we also included in the analyses clinical response (worsening at 1 month vs. no) and CSF malignant cells at 1 month (yes vs. no).

To construct prognostic scores, points were attributed to the final independent prognostic variables from the global OS and 24-week survival Cox models. Global scores were derived for each patient, and 2 or 3 risk categories were defined (i.e., low and high risk or low, intermediate, and high risk). The statistical analyses were performed with MedCalc Statistical Software Version 20.008 (MedCalc Software Ltd., Ostend, Belgium; https://www.medcalc.org; 2021) and GraphPad Prism 10.2.0 (GraphPad software, Boston, MA, USA).

The study was approved by the French National Data Protection Authority and our Institutional Review Board (project number DATA240310; email: CRI-DATA@curie.fr). This study followed the precepts of the Declaration of Helsinki and French laws concerning biomedical research. Per current French regulations, no written informed consent was needed (MR-004 methodology). The project was deposited in the Health Data Hub portal (https://www.health-data-hub.fr/).

## 3. Results

### 3.1. Population

The characteristics of the 109 patients who were diagnosed with MC are depicted in Supporting Table 1. Briefly, 81 patients had HR (hormone receptor)-positive, HER2-negative breast carcinoma (74.3%), 10 out of 109 had HER2+ cancer (9.2%), and 18 out of 109 (16.5%) had TN cancer. Most tumors were of a nonspecific subtype (NST, 66.5%), and 32 out of 109 were infiltrating lobular carcinomas (29.4%).

At MC diagnosis, 44 patients out of 109 (40.4%) had at least one prior or concurrent brain metastasis, and among them, 32 out of 44 (72.7%) had already undergone whole brain radiation therapy. Visceral metastases were present in 67 patients (61.5%). The median number of systemic treatment lines before MC diagnosis was 2 (0–7). The time between initial BC diagnosis and MC diagnosis was significantly longer in HR-positive patients than in HR-negative patients (median time 7 years vs. 2 years, *p* < 0.0001), as was the time between metastasis diagnosis and MC diagnosis (median: 2 years vs. 1 year, *p*=0.004). HER2+ status did not affect the time between the diagnosis of metastasis and MC (median time: 1.8 years vs. 1.8 years, *p*=0.76, when compared to HR + patients). TN status shortened the time between the diagnosis of metastasis and MC (median time to MC metastasis: 11 vs. 25 months, *p*=0.001, when compared to HR-positive patients).

### 3.2. Diagnosis of MC

**Figure 1:**
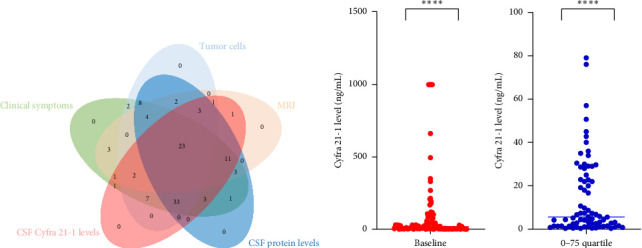
Diagnostic features of meningioma in patients with breast cancer. (a) Venn diagram illustrating the variability of the diagnostic features of meningeal carcinomatosis and how each patient may differ from one another. Each shape represents a specific diagnostic parameter: *green*, clinical symptoms; *light blue*, presence of tumor cells in the cerebrospinal fluid; *light red*, magnetic resonance imaging showing leptomeningeal involvement; *dark blue*, elevated proteins in the cerebrospinal fluid; and orange, elevated Cyfra 21-1 in the cerebrospinal fluid. The figures indicate the number of patients who shared the same diagnostic characteristics. CSF: cerebrospinal fluid; MRI: magnetic resonance imaging. (b) Cyfra 21-1 concentration in the cerebrospinal fluid at diagnosis. *Left, in red*: distribution of all individual values. The blue bar indicates the upper quartile (values > 79 ng/mL). *Right, blue*: details of the Cyfra 21-1 concentration distribution in the first three quartiles.

Most patients (*n* = 102, 93.6%) initially presented with miscellaneous neurological symptoms that triggered further investigation (Table 1 and Figure 1(a)). Brain and/or spinal MRI was initially performed in 92 patients out of 109 (84%). MRI findings were abnormal in 54 out of 92 (59%) patients, and 47 patients out of 54 (87%) with abnormal MRI findings also exhibited MC-related clinical symptoms. Conversely, 38 patients out of 92 (41%) had a normal MRI while presenting with MC symptoms. Seven patients were diagnosed with MR images only. Malignant cells were observed in 85 out of 108 (78.7%) CSF baseline samples. CSF protein levels were abnormal for 101 out of 108 available CSF samples (93.5%) (median value: 1.02 g/mL–normal value < 1 g/mL). We performed CSF dosing with Cyfra 21-1 at diagnosis [9] (Figure 1(b)). The Cyfra 21-1 level (normal value ≤ 1 ng/mL) was elevated in 86 patients out of 95 (90.5%), with a median value of 18 ng/mL (IQR 25–75: 2.8–79). No patient had Cyfra 21-1 elevation as the sole diagnostic feature (Figure 1(a)).

We next analyzed the relationships among the clinical, radiological, and biological diagnostic features (Figure 1(a)) and showed that only 23 patients (29.1% of 79 patients evaluable with all modalities) had a comprehensive MC diagnostic presentation. According to the relationship between MRI and CSF data, 2 patients out of 92 (2.2%) had a diagnosis based on symptoms associated with either elevated CSF protein or Cyfra 21-1 levels but with no CSF malignant cells and normal MRI, while 35 patients out of 71 (49.3%) with CSF malignant cells had a concordant abnormal MRI, and 36 patients out of 71 (50.7%) with CSF malignant cells had a concurrent normal MRI (*p*=0.0016, Fisher's exact test). Overall, 35 of the 53 patients (66.1%) with abnormal MRI findings had malignant CSF cells. Overall, 36 patients out of 38 (94.7%) with a negative MRI had CSF malignant cells, and 18 patients out of 20 (90%) with no CSF malignant cells had an abnormal MRI. Second, exploring the relationship between CSF malignant cells and CSF protein count suggested a correlation trend: 91 patients had elevated CSF proteins, and 73 patients out of 91 (80.2%) had CSF malignant cells (*p*=0.24, chi-squared test). Notably, 10 of 15 patients (66.7%) with a normal CSF protein count had malignant CSF cells. Furthermore, among the 23 patients with no CSF malignant cells, 18 (78.2%) had an elevated CSF protein count. Interestingly, no significant difference was observed between the presence of CSF malignant cells and a baseline elevated CSF protein count (*p*=0.31, Fisher's exact test). Finally, we explored the relationships between the baseline CSF Cyfra 21-1 concentration and the baseline CSF cellularity and CSF protein count. Among the 74 patients with baseline CSF malignant cells, 69 (93.2%) also had elevated CSF Cyfra 21-1. Similarly, among 21 patients with no baseline malignant cells, 17 (81%) had elevated CSF Cyfra 21-1 (*p*=0.10, Fisher's exact test). Likewise, no significant difference was observed between the baseline CSF levels of Cyfra 21-1 and protein (*p*=0.15, Fisher's exact test).

### 3.3. Treatments

We used IT treatments in combination with systemic therapies according to the underlying BC subtype: 88 patients (80.7%) received systemic chemotherapy combined with IT, and 26 patients (23.8%) received endocrine therapy, including 10 patients treated with a CDK4/6 inhibitor. Among the 10 HER2-positive patients, 8 were administered HER2 blocking agents. In 38 patients (out of 44 with concurrent brain metastases, 86.4%), radiation therapy had been previously used to treat brain metastases.

Methotrexate was commonly used as the first-line IT therapy (Supporting Table 2). IT thiotepa was used as a second-line treatment for progressive MC- or methotrexate-related toxicity or when radiotherapy involving the central nervous system (CNS) was indicated [3]. The treatment protocols were established as previously reported [3]. The decision to change the IT treatment was made based on clinical worsening and/or imaging (MRI or scan) of progressive disease. Discontinuation of IT therapies was decided according to clinical worsening when both methotrexate and thiotepa had been used or according to individual quality of life considerations. Malignant CSF cell detection during IT was considered to change or stop IT treatment if concordant with clinical evolution but was not used alone to draw conclusions.

Overall, 103 patients received IT methotrexate, including 97 out of 109 (88.9%) who received it as a first-line treatment. The median number of IT cycles in the overall population was 6 (1–47). IT thiotepa was administered to 42 patients (37.6%), including 12 who received it as a first-line therapy and 29 who received it as a second-line therapy (Supporting Table 2).

### 3.4. Response at One Month

**Figure 2:**
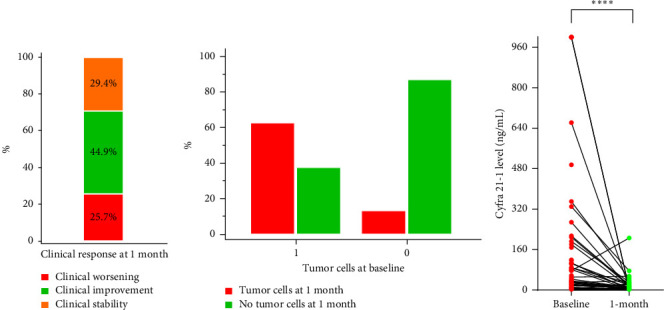
Response evaluation after one month of intrathecal therapy. (a) Clinical response at one month. (b) Variation of tumor cells in the cerebrospinal fluid between baseline (*X* axis; 1 = yes; 0 = no) and 1 month (*Y* axis). (c) Variation in the Cyfra 21-1 concentration in the cerebrospinal fluid between baseline (red dots, each indicating an individual patient) and 1 month (green dots, each indicating an individual patient).

We considered a response to IT therapy at 1 month by neurological clinical evaluation and CSF biology. Clinically, neurological improvement, stability, and worsening were observed in 49 (44.9%), 32 (29.4%), and 28 patients (25.7%), respectively (Figure 2(a)). One-month CSF cytology was evaluable in 87 patients, and almost half of them (*n* = 43, 49.5%) had no detectable tumor cells in the CSF at 1 month, including 28 patients with baseline CSF tumor cells. Among the 17 patients with no CSF malignant cells at baseline, 2 had detectable cells at one month (Figure 2(b)). There was no correlation between clinical and cellular responses (*p*=0.723; Fisher's exact test). We observed a significant decrease in the median CSF Cyfra 21-1 concentration at one month (2.55 ng/mL vs. 18 ng/mL, *p* < 0.0001; Wilcoxon rank sum test, Figure 2(c)). We again found no relationship between a decrease in the Cyfra 21-1 level and clinical response (*p*=0.856; ANOVA and chi-squared test).

### 3.5. Progression-Free Survival and OS

The median follow-up was 57.5 months (45–ongoing). The five-year survival rate was 1.8%. Age and baseline performance status were not related to OS. The median OS was 32.4 weeks (range 2.6–230). By univariate analysis (Supporting Table 3), 1-month clinical response was the only parameter associated with OS, whether considered a two-class (worsening vs. no) or three-class (worsening, stability, and improvement) variable. The TN subtype (*p*=0.0675) and a baseline CSF Cyfra 21-1 threshold value > 79 ng/mL (i.e., in the upper quartile; *p*=0.0544; C-index = 0.538) attained borderline significance and were therefore retained in the multivariate analyses. Treating the Cyfra 21-1 value as a continuous variable did not improve the model performance. Due to the very poor prognosis of patients with MC, we also examined 24-week survival. In addition to the 1-month clinical response, which was also associated with 24-week survival, the number of previous lines of therapy, a baseline CSF Cyfra 21-1 value below the median (18 ng/mL), and the absence of tumor cells at 1 month in the CSF also attained borderline significance (*p* < 0.1) and were retained in the multivariate analyses. We performed two Cox proportional hazards regression analyses (Table 2). Clinical response at 1 month and a baseline CSF Cyfra 21-1 value > 79 ng/mL were independently associated with OS. Clinical response at 1 month and the absence of tumor cells in the CSF at 1 month were associated with 24-week survival. The proportionality of the Cox models was ensured by Schoenfeld residuals (Supporting Figure 1).

In addition, we examined progression-free survival (Supporting Table 3). The median PFS was 27.1 weeks (range 1.6–174.0). No clinical or baseline parameters were significantly associated with PFS. We did not evaluate the relationship between PFS and response-related parameters. We also did not look at the duration of response, a surrogate of progression-free survival, due to the overall poor prognosis of leptomeningeal carcinomatosis patients.

### 3.6. Prognostic Score

**Figure 3:**
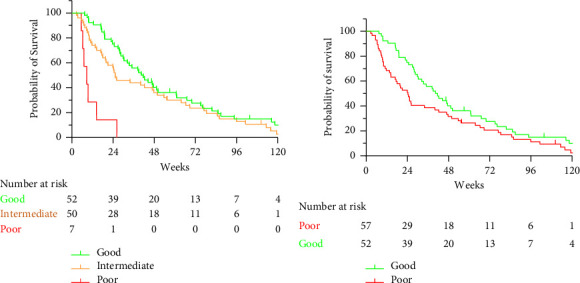
Overall survival. (a) Overall survival according to the 3-class prognostic score. (b) Overall survival according to a 2-class prognostic score.

To develop a clinically actionable prognostic tool, we constructed different scores based on the results of the Cox models (Table 2). For each outcome (overall or 24-week survival), we were able to construct 2 or 3 groups of patients with distinct prognoses. The 2-class scores were constructed as follows. Patients were classified in the “good prognosis” group when both variables were favorable (0 points), and alternatively, they were classified in the “poor prognosis” group (1 or 2 points). To construct the 3-class scores, patients were classified in the “good prognosis” group when both variables were favorable (0 points), in the “poor prognosis” group when both variables were unfavorable (2 points), and in the “intermediate prognosis” group when either variable was favorable (1 point). The results are depicted in Figure 3 and Supporting Figure 2. In terms of OS, the 3-class score based on 1-month clinical response and baseline CSF Cyfra 21-1 identified clinically and significantly different survival rates, notably for the patients with poor prognosis who all died at 24 weeks and had a median survival of approximately 8 weeks (Figure 3(a)). The median OS of each group is detailed in Table 2. We specifically examined 81 patients with luminal BC, and the results were very similar (Supporting Figure 3).

These results prompted us to specifically look at 24-week survival, a reasonable clinical endpoint in this clinical setting. Short-term survival was closely related to 1-month response parameters according to both clinical and biological evaluations. Both the 3-class (Supporting Figure 2a) and 2-class (Supporting Figure 2b) groups were highly significantly associated with short-term survival. Notably, all 17 patients with both unfavorable response variables at 1 month (clinical response and CSF malignant cells) died at 18 weeks.

## 4. Discussion

Here, we report important observations about the diagnosis, treatment, and prognosis of MC in patients with advanced BC. Our cohort appears representative of this clinical setting, with an important proportion of patients having very advanced BC, lobular carcinoma, or TN cancer subtypes, as previously reported [1, 8, 11]. Of note, results have been reported in the preprint format (https://doi.org/10.21203/rs.3.rs-3979871/v1) and are further discussed here.

MC may frequently be clinically misleading. Here, we describe how a multidimensional approach may be necessary to confirm the diagnosis of MC, beyond the EANO criteria [6]. Of note, these criteria have been recently updated and completed, underlining the need for a comprehensive approach combining a thorough clinical evaluation together with brain imaging and CSF analyses [6, 12]. Almost all our patients underwent the three procedures. Our observations are similar to those in the literature, with elevated protein levels in the CSF (> 50 mg/dL) in 56%–91% of patients [5]. The diagnostic relevance of Cyfra 21-1 has been confirmed, as elevated CSF levels of Cyfra 21-1 are associated with all other diagnostic features. This result is similar to those of several studies on MCs from other primitive solid tumors, such as lung cancer [13] or head and neck cancer [14]. Abnormal baseline Cyfra 21-1 did not reach statistical significance for prognosis, although evidence suggests that after the start of IT treatment, elevated CSF Cyfra 21-1 is an independent adverse prognostic factor [3]. Statistical significance was reached for a baseline CSF Cyfra 21-1 value greater than 79 ng/mL, corresponding to patients in the upper quartile, which we used in our study. Overall, we showed that fewer than 25% of patients had comprehensive features of MC (Figure 1(a)). Taken together, these results suggest that the formal diagnosis of MC should rely on a combinatorial but not exclusive approach, harnessing clinical, biological, and radiological methods.

We used widely described therapies for patients with advanced BC, including IT [1, 3, 6, 9]. We focused the present analysis on early response to IT therapy and OS to identify which patients could benefit from extended IT. After one month of IT treatment, almost all patients experienced either an improvement in clinical symptoms, which triggered early clinical evaluation as a potential prognostic factor, or a decrease in the presence of tumor cells in the CSF or in the Cyfra 21-1 CSF level, with any relevant correlation, in line with the current limitations in evaluating the response in leptomeningeal disease [7]. Only 15 patients had a comprehensive response, none of whom were evaluable with magnetic resonance imaging. Notably, to date, no standardized MRI evaluation of leptomeningeal disease has been developed due to very low interobserver agreement [7].

Based on these results, we performed prognostic univariate and multivariate analyses on individual response criteria. The median OS was 32.4 weeks (range 2.6–230), consistent with recent large-scale studies [8]. Most interestingly, clinical evaluation at one month was the most statistically significant factor associated with OS and 24-week survival. Clinical response at one month retained its powerful prognostic value in all multivariate models. Specifically, treating patients with IT has been demonstrated to improve progression-free survival and reduce the number of MC-related death events [4]. Other biologically sound parameters also attained borderline significance, such as TN subtype (which was not retrieved from the analyses), CSF Cyfra 21-1 levels, or the presence of tumor cells, and were retained in the multivariate models. None of the features associated with cancer history or disease burden showed significant prognostic value, as opposed to previous studies that did not consider either early clinical evolution or key MC-specific biological parameters [1, 9, 15].

Several scores have already been proposed, including in very recent studies, to refine the prognostic evaluation of MC; however, these scores have very limited discriminating properties [8]. Here, we introduce two important novelties in the construction of four original prognostic scores. First, we based the scores on a combination of clinical evaluation and biological response criteria specific to MC. Second, we considered both OS and 24-week survival because of the poor prognosis of BC patients with MC and for clinical relevance purposes. Regarding OS, the 3-class score based on 1-month clinical response and baseline Cyfra 21-1 (in the upper quartile) identified a subset of patients with a very poor prognosis. Regrouping the poor and intermediate prognosis groups confirmed the clear identification of two different prognostic subgroups. The 24-week OS prognostic score also indicated a very poor prognosis. This result appears clinically relevant and applicable, as it is based on two easily measurable variables in daily practice, namely, clinical response and 1-month CSF tumor cell count, whereas Cyfra 21-1 is not always used for biological analyses.

The present study has several limitations. This was an observational, retrospective, and monocentric study, with a limited sample size. Due to our very specific approach combining the evaluation of neurological clinical response by the same trained clinical team and original biological CSF analyses, it was not possible to construct a validation cohort. However, as we continue to treat BC patients who suffer from MC with the same protocols, a validation cohort will be constructed after a sufficient follow-up. Notably, our population consisted exclusively of patients treated with IT, mostly with methotrexate limiting treatment heterogeneity. This may be a recruitment bias because only patients who could be treated at the onset of MC were selected; thus, the data do not represent the entirety of patients with advanced BC and MC. We also cannot draw any conclusions with regard to the HER2-positive subset of patients, who were treated before the modern era of HER2 targeted therapies which may have benefit on leptomeningeal disease such as antibody–drug conjugates.

In summary, and to the best of our knowledge, this report is the first to propose comprehensive prognostic scores for both long- and short-term OS in patients with advanced BC and MC who are treated with IT chemotherapy. As there is still no standard of care for such patients, we suggest that simple features such as clinical evaluation, the presence of tumor cells in the CSF after one month of treatment, and the baseline CSF level of Cyfra 21-1 could be used to adequately determine which patient will potentially benefit from prolonged IT.

## Figures and Tables

**Table 1:** Diagnosis of meningeal carcinomatosis.

	*n*	%
*WHO performance status N = 109*		
0	89	81.6
1	17	15.6
2	3	2.7

*MC symptoms N = 109*		
Symptomatic	102	93.6
Asymptomatic	7	6.4

*Most frequent MC symptoms N = 102*		
Dizziness	28	27.5
Headache (and no intracranial hypertension syndrome)	27	26.5
Intracranial hypertension syndrome (nausea, vomiting, headache)	24	23.5
Cranial nerve dysfunction	17	16.7
Confusion	15	14.7
Motor paralysis	11	10.8
Sensory symptoms	11	10.8
Meningeal syndrome (headache + neck stiffness + phono or photophobia)	5	4.9

*Malignant cells N = 108*		
Yes	85	78.7
No	23	21.3

*CSF protein level at baseline N = 108*		
Normal	7	6.5
Elevated	101	93.5

*CSF level of Cyfra 21-1 at baseline N = 95*		
Normal	9	9.5
Elevated	86	90.5

*MC according to EANO-ESMO criteria*		
Positive cytology	85	77.9
Probable diagnosis (MRI abnormalities and neurological symptoms)	47	43.1
Possible diagnosis (either MRI abnormalities or neurological symptoms)	62	56.9

Abbreviations: CSF, cerebrospinal fluid; MC, meningeal carcinomatosis.

**Table 2:** Results of the Cox proportional hazards regression analyses.

	Categories	HR (95% CI)	*p* value
*Overall survival*			
1-month clinical Response	A⁣^∗^ + B⁣^∗∗^ C⁣^∗∗∗^	1^§^ 2.00 (1.23–3.28)	0.006
Baseline CSF Cyfra 21-1 value	≤ 79 ng/mL > 79 ng/mL	1 1.73 (1.07–2.80)	0.025

*24-week survival*			
Clinical response	A⁣^∗^ + B⁣^∗∗^ C⁣^∗∗∗^	1 5.96 (3.05–11.37)	< 0.0001
1-month CSF Tumor cells	No Yes	1 2.28 (1.16–4.50)	0.017

Abbreviations: 95% CI, 95% confidence interval; HR, hazard ratio.

^§^1 denotes the reference category.

⁣^∗^A: improvement.

⁣^∗∗^B: stability.

⁣^∗∗∗^C: worsening.

## Data Availability

The data will be made available upon reasonable request to the corresponding author.

## Supporting Information

Supporting Table 1: population characteristics at the diagnosis of meningeal carcinomatosis. This table lists the main clinical characteristics of the population, at the diagnosis of meningeal carcinomatosis.

Supporting Table 2: details of intrathecal therapies. This table shows how intrathecal therapies were used, in the whole population, and by subtype.

Supporting Table 3: overall survival, 24-week survival, and progression-free survival univariate analyses. This table shows the results of the univariate analyses for overall survival, 24-week survival, and progression-free survival. All relevant potential prognostic features were used.

Supporting Figure 1: Schoenfeld residuals of the final Cox proportional hazard model. For the three final variables, it is shown that the scaled Schoenfeld residuals are centered over time about a horizontal line at zero. No time dependence is suggested, underlining the proportionality of the model.

Supporting Figure 2: 24-week survival. This figure shows the 24-week survival curves, based on the 3-class and 2-class prognostic scores.

Supporting Figure 3: overall survival in patients with luminal breast cancer, according to the 3-class composite prognostic score. This figure shows the overall survival curve in the subset of patients with luminal breast cancer, according to the 3-class prognostic score.
