# Supplementary material for: Development of a Prognostic Score in Patients With Advanced Breast Cancer Treated for Meningeal Carcinomatosis
Source: Breast J. 2025 Mar 7;2025:5438600. doi: 10.1155/tbj/5438600 (PMC11991760; doi:10.1155/tbj/5438600)
Supplement: Supporting Information — Additional supporting information can be found online in the Supporting Information section. [file 5438600.f1.docx]

**Supplementary table 1: population characteristics at the diagnosis of meningeal carcinomatosis**

|  | | **N (%)** |
| --- | --- | --- |
| **Gender** | |  |
| Women | | 108 (99.1%) |
| Men | | 1 (0.9%) |
| **Biological subtype** | |  |
| HR+/HER2neg | | 81 (74.3%) |
| HER2 positive | | 10 (9.2%) |
| Triple negative | | 18 (16.5%) |
| **Pathological subtype** | |  |
| NST* | | 70 (64.2%) |
| Lobular | | 32 (29.4%) |
| Other | | 6 (5.5%) |
| Visceral metastases | | 67 (61.5%) |
| Liver metastases | | 61 (91.0%) |
| Other (without liver metastases) | | 6 (8.9%) |
| **Brain metastasis at MC diagnosis** | 44 (40.4%) |  |
| Prior CNS radiation therapy | 32 (72.7%) |  |
| **Number of systemic therapy lines before MC** |  |  |
| 0 | 21 (19.3%) |  |
| 1 | 15 (13.8%) |  |
| 2 | 24 (22.0%) |  |
| 3 | 17 (15.6%) |  |
| 4 | 18 (16.5%) |  |
| 5 and more | 14 (12.9%) |  |

**NST: non-specific type*

**Supplementary table 2: Details of intrathecal therapies**

|  | **HR+**  **N=81** | **HER2+**  **N=10** | **TNBC**  **N=18** | **All patients**  **n=109** |
| --- | --- | --- | --- | --- |
| **Methotrexate**  **(median 6, range 0-47)** | 76 (93.8%) | 10 (100%) | 17 (94.4%) | 103 (94.5%) |
| **Thiotepa**  **(median 4, range 0-8)** | 32 (39.5%) | 3 (30%) | 7 (38.9%) | 42 (11.0%) |
| **IT therapy (L1)** | 82* (100%) | 10 (100%) | 18 (100%) | 110* (100%) |
| **IT therapy (L2)** | 26 (32.1%) | 3 (30%) | 6 (33.3%) | 35 (32.1%) |

**One patient received methotrexate and thiotepa as part of the first line of IT therapy*

**Supplementary table 3**: **Overall survival, 24-weeks survival and progression free survival univariate analyses**

|  | **Categories** | **Overall survival**  **HR (95% CI)** | | **24-weeks survival**  **HR (95% CI)** | | **Progression free survival**  **HR (95% CI)** | |
| --- | --- | --- | --- | --- | --- | --- | --- |
| **Age** | ≤ 65y  <65y | 1^§^  1.31 (0.81-2.11) | P=0.266 | 1  1.09 (0.45-2.65) | P=0.839 | 1  1.60 (0.92-2.77) | P=0.092 |
| **WHO performance status** | 0  1-2 | 1  1.28 (0.79-2.45) | P=0.302 | 1  1.12 (0.57-2.81) | P=0.653 | 1  1.06 (0.65-2.32) | P=0.524 |
| **Visceral metastases** | No  Yes | 1  1.30 (0.85-1.99) | P=0.218 | 1  1.49 (0.75-2.96) | P=0.255 | 1  1.02 (0.63-1.63) | P=0.951 |
| **Brain metastases** | No  Yes | 1  1.02 (0.69-1.52) | P=0.916 | 1  1.07 (0.57-2.01) | P=0.826 | 1  1.02 (0.67-1.58) | P=0.919 |
| **Number of previous lines** | 0-2  3+ | 1  1.04 (0.71-1.55) | P=0.82 | 1  1.68 (0.91-3.11) | P=0.096 | 1  0.88 (0.57-1.35) | P=0.558 |
| **Triple negative subtype** | No  Yes | 1  1,81 (0,96-3,40) | P=0.0675 | 1  1.23 (0.54-2.79) | P=0.625 | 1  0.93 (0.48-1.79) | P=0.835 |
| **Neurological symptoms at baseline** | No  Yes | 1  1.17 (0.57-2.41) | P=0.674 | 1  1.21 (0.33-4.43) | P=0.768 | 1  1.29 (0.58-2.89) | P=0.524 |
| **CSF tumor cells** | No  Yes | 1  0.76 (0.46-1.28) | P=0.305 | 1  1.36 (0.65-2.83) | P=0.418 | 1  0.81 (0.46-1.42) | p=0.457 |
| **CSF proteins** | Normal  Elevated | 1  0.81 (0.45-1.47) | P=0.489 | 1  1.58 (0.68-3.66) | P=0.287 | 1  1.01 (0.54-1.88) | P=0.968 |
| **Baseline CSF Cyfra 21-1** | Normal  Elevated | 1  1.45 (0.78-2.69) | P=0.234 | 1  2.41 (0.89-6.51) | P=0.083 | 1  1.72 (0.89-3.29) | P=0.105 |
| **Baseline CSF Cyfra 21-1 value** | ≤ 79 ng/mL  > 79 ng/mL | 1  1.68 (0,99-2,84) | P=0.0544 | 1  1.61 (0.77-3.35) | P=0.203 | 1  1.24 (0.71-2.17) | P=0.445 |
| **Baseline CSF Cyfra 21-1 > median value** | No  Yes | 1  1.31 (0.86-2.00) | P=0.204 | 1  1.80 (0.95-3.42) | P=0.070 | 1  1.26 (0.79-1.99) | P=0.330 |
| **1-month clinical response** | A*  B**  C*** | 1  1.23 (0.79-1.93)  1.85 (1.07-3.18) | P=0.036 | 1  1.57 (0.79-3.15)  6.29 (2.60-15.20) | P<0.0001 | - |  |
| **1-month clinical response** | A + B  C | 1  1.93 (1.14-3.28) | P=0.015 | 1  13.38 (5.69-31.45) | P<0.0001 | - |  |
| **1-month CSF tumor cells** | No  Yes | 1  1.26 (0.85-1.87) | P=0.245 | 1  2.08 (1.12-3.85) | P=0.019 | - |  |
| **1-month Cyfra 21-1** | Nomal  Elevated | 1  1.09 (0.63-1.90) | P=0.76 | 1  1.11 (0.39-3.19) | P=0.845 |  |  |

**A : improvement; **B : stability; ***C: worsening. HR: hazard ratio. 95% CI: 95% confidence interval. ^§^1 denotes the reference category.*

**
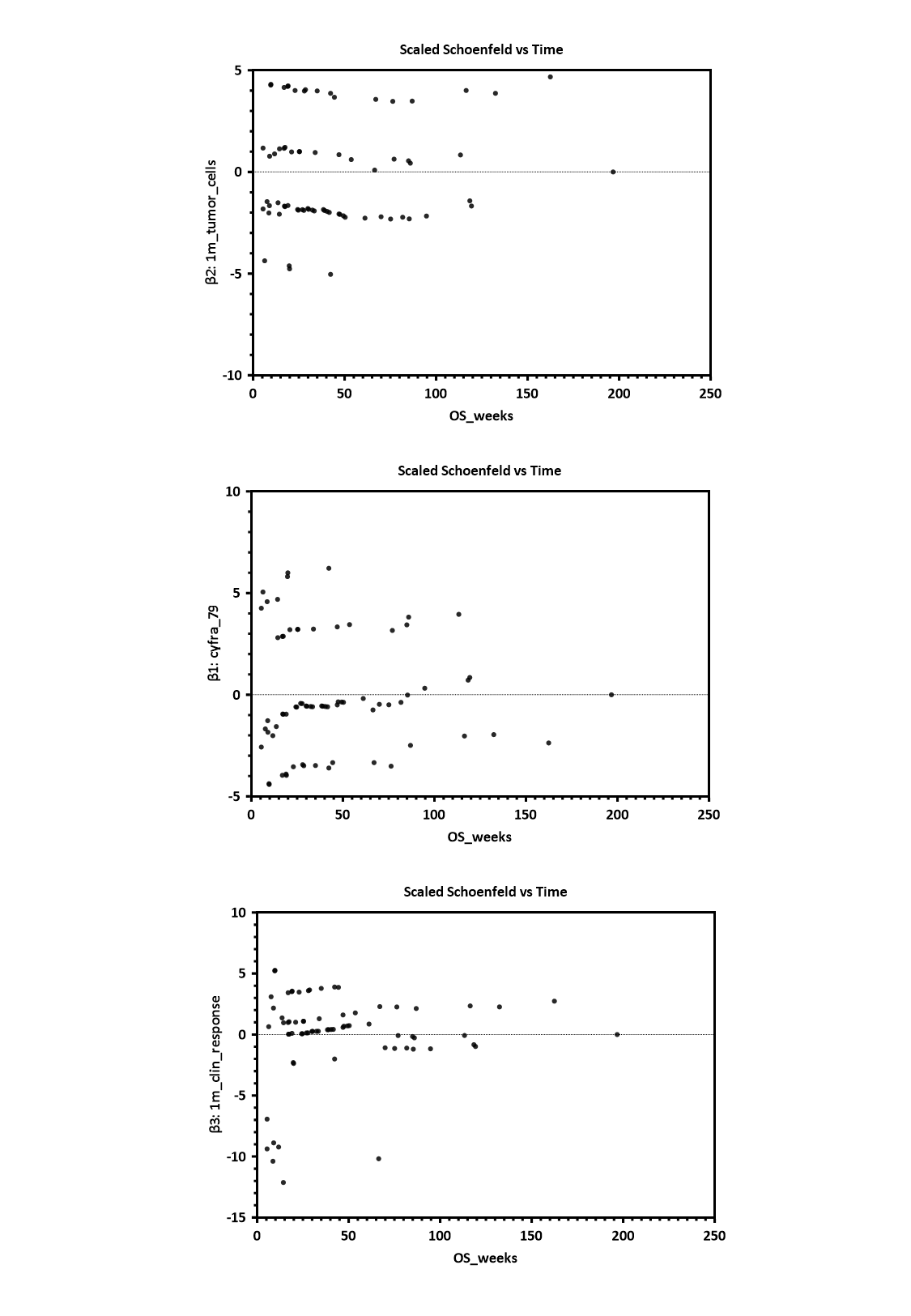
Supplementary Figure 1. Schoenfeld residuals of the final Cox proportional hazard model.**

**Supplementary Figure 2. 24-weeks survival**

1. 24-weeks survival according to a 3-class prognostic score
2. 24-weeks survival according to a 2-class prognostic score


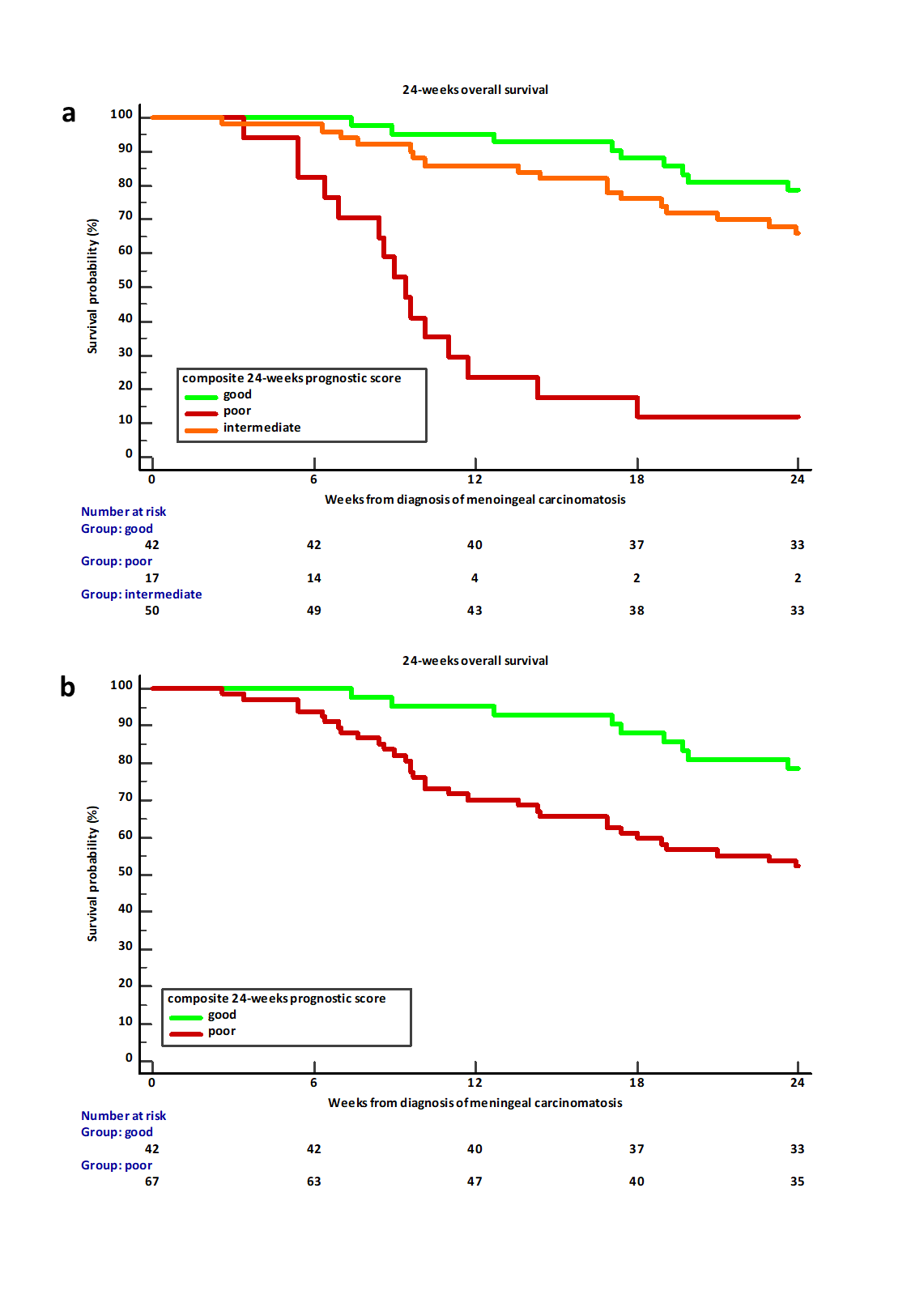


**Supplementary Figure 3. Overall survival in patients with luminal breast cancer, according to the 3-class composite prognostic score.**
